# Reducing toxic constituents of ginkgolic acid content and improving bioactive flavonoid content from *Ginkgo biloba* leaves by high‐temperature pretreatment processing

**DOI:** 10.1002/fsn3.3118

**Published:** 2022-10-30

**Authors:** Fen Zhao, Shan Huang, Liufeng Ge, Yongzhen Wang, Yuwei Liu, Cunshe Chen, Xinqi Liu, Qianwen Han

**Affiliations:** ^1^ Beijing Advanced Innovation Center for Food Nutrition and Human Health, Beijing Engineering and Technology Research Center of Food Additives Beijing Technology and Business University (BTBU) Beijing China; ^2^ Beijing Harmony Health Medical Diagnostics Co., Ltd. Beijing China; ^3^ Inner Mongolia Xibei Restaurant Group Co., Ltd. Beijing China; ^4^ Beijing Science Sun Pharmaceutical Co., Ltd. Beijing China; ^5^ Zhongbai Xingye Food Technology (Beijing) Co., Ltd. Beijing China

**Keywords:** antioxidant activities, flavonoids, *Ginkgo biloba*, ginkgolic acids, high‐temperature pretreatment, response surface methodology, UPLC‐QTOF‐MS/MS

## Abstract

High‐temperature pretreatment was developed in this article to remove the main toxic constituents of ginkgolic acids (GAs) from *Ginkgo biloba* leaves (GBLs) and improve the bioactive flavonoid content by water extraction. To optimize the effects of high‐temperature pretreatment process parameters on removing toxic GAs to a limited level and improving the content of bioactive flavonoids, a Box–Behnken design (BBD) combined with response surface methodology (RSM) was also conducted. The results showed that the content of GAs could be reduced to 4.11 ppm and the highest content of flavonoids could reach 3.51% under the optimized conditions of high‐temperature pretreatment process of 177°C with water extraction at 96°C at a liquid‐to‐solid ratio of 56:1. The content of toxic GAs substantially decreased by 83.50% while the content of bioactive flavonoids increased by 44.30% compared with the conventional water extraction method. Moreover, the new process was more efficient, environmentally friendly, and could get avoid a subsequent multi‐step process of removing toxic GAs. The crude extracts were then purified by macroporous resin to obtain the 60% ethanol fraction. After purification, the flavonoid content increased to 43.50% while the GAs were not detected. The main compounds of 60% ethanol fraction were identified by UPLC‐QTOF‐MS/MS. Antioxidant activities including reducing powder, 2,2‐diphenyl−1‐picrylhydrazyl (DPPH) radical scavenging, and OH^·^ scavenging assays all showed that the 60% ethanol fraction was better than the butylated hydroxytoluene (BHT) standard.

## Graphical abstract

High temperature pretreatment processing was used in the article to reduce toxic constituents of ginkgolic acids content and improve bioactive flavonoids content from Ginkgo biloba leaves. Box‐Behnken design combined with Response surface methodology was conducted to obtain the optimal conditions. The 60% ethanol purification fraction was further analyzed by UPLC‐QTOF‐MS/MS and antioxidant activities all showed better abilities than the BHT standard.

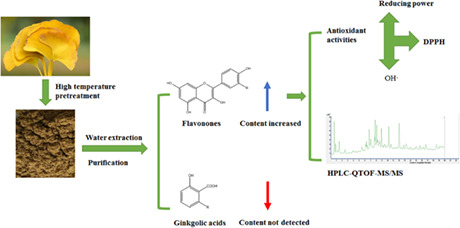

## 1 INTRODUCTION

**FIGURE 1:**
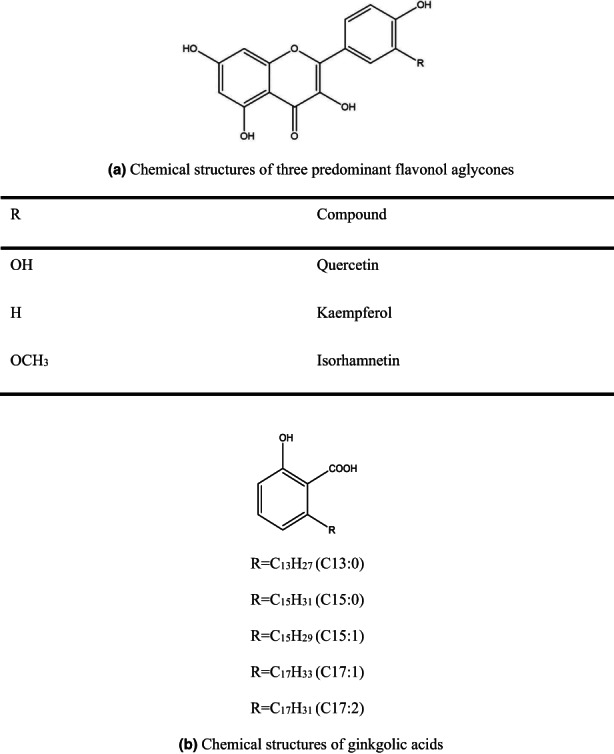
Chemical structures of three predominant flavonol aglycones (a) and ginkgolic acids (b) in *Ginkgo biloba*.

*Ginkgo biloba* L., the sole surviving species in the family Ginkgoaceae, is commonly known as a “living fossil” (Strømgaard & Nakanishi, 2004). This Archean plant is native to China, but has been cultivated worldwide for its horticultural, timber, and medicinal properties. GBLs contain various species of active ingredients, such as ginkgolides, bilobalide, flavonoids, proanthocyanidins, alkylphenols, and simple phenolic acids (Beek, 2002). Most of the *Ginkgo biloba* extract (GBE) were made from GBLs, which have two types, namely full extracts and standardized extracts. GBE‐containing flavonoids are one of the most popular herbal supplements widely used for medicaments, foods, and cosmetics. Pharmaceuticals derived from GBE have been used to treat tinnitus, cognitive impairment and Alzheimer's disease, retinal, cardiovascular, ischemia cerebrovascular, peripheral vascular, diabetic nephropathy, and other diseases (Liu et al., 2021). Among the various ingredients of GBLs, flavonoids including flavones, flavonol glycosides, biflavones, proanthocyanidins, and isoflavonoids are one of the most important active ingredients in *G. biloba*. Flavonoids exhibited remarkable effects, including antioxidation, anticancer, antibacterial, antiviral, antiinflammatory, and neuroprotective effects (Wang et al., 2016; Yan & Wu, 2016; Zhang et al., 2018, 2019). Hydrolysates of flavonol glycosides contain three predominant flavonol aglycones, that is, quercetin, kaempferol, and isorhamnetin (Chen et al., 2010; Figure 1a). Meanwhile, GAs were the toxic ingredients, which have cytotoxic potential and can induce neuronal death and activate protein phosphatase type 2C (Ahlemeyer et al., 2001). Therefore, the domestic and foreign pharmacopeias require that the GAs content in the standard GBE should be lower than 5.00 mg/kg (Dong et al., 2021).

There are many extraction methods developed to obtain flavonoids in *G. biloba*, such as water extraction, organic solvent extraction, heat reflux extraction, Soxhlet extraction, microwave‐assisted extraction, supercritical fluid extraction, enzymes‐assisted extraction (Liu et al., 2015a), ultrasonication, membrane adsorption, molecular imprinting (Chen et al., 2011), and prefermentation treatment (Wang et al., 2013). However, only ethanol/acetone is generally used in commercial processes. And, complicated processes such as purification and organic solvent extraction were subsequently needed in order to further remove the toxic component of GAs, which will cause higher costs, residual solvents, and loss of flavonoids. Thus, considering the bioactivities of flavonoids and toxicities of GAs, developing new methods that can improve the content of bioactive flavonoids while reducing the content of toxic component of GAs effectively were desperately needed in the food industry. The water solubility of GAs is very poor; considering the cost and environmental protection, water was selected to be the extraction solvent in this study. However, considering the solubility of other bioactive constituents, such as flavonoids, only water extraction could not guarantee the extraction rate. Furthermore, only water extraction may not limit the content of GAs to the requirements of Chinese Pharmacopeia (2020) and Europe Pharmacopeia 9.0 (2011), and the subsequent purification was still needed, which will cause higher cost, residual solvents, and loss of flavonoids. Based on the above, another method was still needed together in order to reduce the content of GAs while improving flavonoid content.

GAs are mixtures of a series of GA homologs (Figure 1b). They possess 13–17 long‐chain hydrophobic bases at site 6 of alkyl salicylic acid and 0–3 side‐chain double bonds (Beek, 2002; Beek & Montoro, 2009; Zhu et al., 2018). Yang et al. (2014) have reported that the GAs were stable at 50°C or below. However, when the temperature reached 70°C, the GAs underwent thermal decomposition. The thermal decomposition derivatives of the GAs under 250°C are ginkgoales; of which ginkgol C17:1 has the strongest inhibition migration on SMMC‐7721 cells in a dose‐ and time‐dependent manner. Based on the above, high temperature could help to reduce the content of GAs.

Except for the toxic GAs, there are other bioactive constituents in *G. biloba*, such as flavonoids and terpene lactones. Zhao (2011) have reported that rutin was basically undecomposed when the heated temperature was below 160°C, and between 160°C and 200°C, rutin partially decomposed into quercetin. When over 210°C, rutin was all damaged. The literature showed that terpene lactones would decompose in the temperature range 280–430°C (Lü et al., 2011).

Based on the above, in this study, the toxic components of GAs were expected to be partially degraded with high‐temperature pretreatment while the bioactive components of flavonoids and terpene lactones were not to be broken down by controlling the high temperature. A BBD combined with RSM was conducted to optimize the effects of process parameters on the content of bioactive flavonoids. Then, macroporous resin was used to purify the water extracts and the main compounds were identified by UPLC‐QTOF‐MS/MS. The antioxidant activities of the extracts were examined by reducing power, DPPH radical scavenging, and OH^·^ scavenging assays.

## 2 EXPERIMENTAL PROCEDURES

### 2.1 Materials

*Ginkgo biloba* leaves used in this study were bought from Beijing Tongrentang Group Corporation and identified by Prof. Li Li (Beijing Technology and Business University). D380 macroporous adsorbent resin was obtained from Nankai University Chemical Plant. Rutin standard was purchased from the National Institutes for Food and Drug Control. BHT standard was purchased from TCI Development Co. Ltd. Glucose and total GAs standards were purchased from Beijing Tetrahedron Bio. Ltd. DPPH^·^ was purchased from TCI Development Co. Ltd. Iron (III) potassium ferricyanide (K_3_Fe(CN)_6_), trichloroacetic acid (TCA), ferric chloride (FeCl_3_), ferrous sulfate (FeSO_4_), hydrogen peroxide (H_2_O_2_), and phosphate buffer were obtained from Xilong Scientific Co. Ltd. All other solvents and chemicals were obtained from China National Pharmaceutical Group Co. Ltd. and were of analytical grade.

### 2.2 High‐temperature pretreatment and single‐factor conditions

Dried *G. biloba* leaves of 4.0 g were heated in the drying oven at different high temperatures for 1 h. Then, the leaves were separately grounded into powders, followed by sieving through a 60 mesh screen of 0.25 mm to control the particle size. The powders were extracted with water at different liquid‐to‐solid ratios for 3.5 h at different extraction temperatures. The extracts were filtered, and the solvent was evaporated at 50°C under vacuum. Although GAs could be decomposed at a high temperature, the flavonoids in *G. biloba* leaves may also be decomposed. For example, rutin could not be decomposed when the temperature was below 160°C. However, it begins to partly decompose to quercetin when the temperature is higher than 160°C and completely decomposed when the temperature is higher than 210°C. Thus, the levels of high‐temperature factor were 100, 120, 140, 160, 180, and 200°C. In our preexperiment, we extracted three times and found that both flavonoids and GAs were extracted more at the second and third times. However, once extraction could greatly improve the content of flavonoids compared with the traditional method and the GAs were also correspondingly less, we extracted. Minitab 17 software was used to research five factors: extraction time, extraction temperature, liquid‐to‐solid ratio, pretreatment temperature, and pretreatment time. And, we found the influence of factors was extraction temperature > liquid‐to‐solid ratio > pretreatment temperature > pretreatment time > extraction time. Thus, we selected the three most influential factors. That is, extraction temperature, liquid‐to‐solid ratio, and pretreatment temperature. Based on our preexperiment and the references, the levels of extraction temperature factor were 50, 60, 70, 80, 90, and 100°C and the levels of liquid‐to‐solid ratio factor were 40:1, 45:1, 50:1, 55:1, 60:1, and 65:1. Three factors of high‐temperature pretreatment (100, 120, 140, 160, 180, and 200°C), extraction temperature (50, 60, 70, 80, 90, 100°C), and liquid‐to‐solid ratio (40:1, 45:1, 50:1, 55:1, 60:1, 65:1) were considered to be the most important in this extraction process.

### 2.3 Bioactive flavonoid content of water extracts

The UV–vis spectrophotometry method described by Chen et al. (2014) was used to determine the bioactive flavonoid content of water extracts. A standard solution (0.1 mg/ml) of rutin was prepared. The solvent used in this process was ethanol–water (60:40, v/v). And then, 2.0, 3.0, 4.0, 5.0, 6.0, and 7.0 ml rutin solutions were removed in six volumetric flasks (10 ml). Next, adding 0.15 ml of 5% NaNO_2_ solution, 0.15 ml of 10% Al(NO_3_)_3_ solution, and 2 ml 4% NaOH solution in sequence, and waiting for 5 min after each addition, then followed by adding ethanol–water (60:40, v/v) solvent to the scale. The sample solution without coloration was used as a reference. UV–vis spectrophotometer of Agilent Cary 60 was used with the determination wavelength of 510 nm to test absorbance. Results were used to draw the rutin standard curve.

One milliliter of the extracts was removed and diluted to 10 ml in a volumetric flask (10 ml) by ethanol–water (60:40, v/v) solvent, and colorated and analyzed using the method stated above. The content (%) could be calculated as Equation (1).

(1) $$\text{Content}=\left(C\times n\times V\right)/M\times 100\%$$

where *C* (mg/ml) was the flavonoid concentration calculated by rutin standard curve, *V* (ml) was the volume of the extracts, *n* was the dilution ratio, and *M* (g) was the mass of water extracts.

### 2.4 Experimental design and statistical analysis

In the present study, we used Design Expert Version 8.0.6.1 software as a design and analysis tool to conduct experiments. Regression coefficients, the significance of the process variables, conformity of the experimental data to models, and optimal response variables can be obtained by using this software.

A three‐factor RSM and BBD were applied for determining optimal extraction process conditions. Pretreatment temperature (*A*: 160–200°C), liquid‐to‐solid ratio (*B*: 50:1–60:1), and extraction temperature (*C*: 80–100°C) were chosen as independent or process variables, while response variable was the content of flavonoids. After optimization, each variable was coded at three levels −1, 0, and +1 as shown in Table 1.

**TABLE 1:** Factors and levels of the Box–Behnken test design

Factors	Levels		
	−1	0	1
Pretreatment temperature (*A*)	160	180	200
Liquid‐to‐solid ratio (*B*)	50:1	55:1	60:1
Extraction temperature (*C*)	80	90	100

The accuracy of the model was investigated by the regression analysis (*R* ^2^). An *F*‐test was conducted to analyze the significance of the model terms. The response surface plots and contour plots were used in combination to show how the process variables affect the response variables.

### 2.5 Optimization of enrichment parameters of macroporous resin and purification of water extracts

Three macroporous adsorbent resins D380, DA201, and AB‐8 were selected to further purify the water extracts and enrich the bioactive flavonoids. Static adsorption, dynamic adsorption, and loading quantity were studied. 2.0 g macroporous adsorbent resin was weighed and put into 100‐ml conical flask. 40 ml known flavonoid concentration of extracting solution was added and put in gas bath thermostatic oscillator; the temperature was 29.8°C, the rotation 120 rpm, and oscillated and adsorbed for 24 h. The adsorptive capacity was tested. Then, the saturated resin was washed several times with water and put in a 100‐ml triangular flask; and 30 ml 70% ethanol solution was added and put in a gas bath thermostatic oscillator. The temperature was 29.8°C, the rotation was 120 rpm, and oscillated and adsorbed for 24 h. After filtering, the mass concentration of flavonoids was tested. The adsorbing capacity, adsorption rate, and desorption rate were studied. The adsorbing capacity (*Q*, mg/g resin) was calculated as shown in Equation (2), the adsorption rate (*E*, %) was calculated as Equation (3), and the desorption rate (*D*, %) was calculated as Equation (4).

(2) $$Q=\left(C_{0}-C_{e}\right)V_{i}/W$$

(3) $$E=\left(C_{0}-C_{e}\right)/C_{0}$$

(4) $$D=C_{d}V_{d}/\left(C_{0}-C_{e}\right)V_{i}\times 100\%$$

where *C*_0_ (mg/ml) was the initial concentration of the solution, *C*_e_ (mg/ml) was the equilibrium concentration of the solution, *V*_*i*_ (mg/ml) was the adsorption equilibrium concentration of the solution, *W* was the resin weight, *C*_d_ (mg/ml) was the concentration of desorption solution, and *V*_d_ (mg/ml) was the volume of the desorption solution.

The concentration of flavonoids in the eluent was tested using a UV spectrophotometer every 2 min during elution. If the concentration reached 10% of the loading concentration, the column was saturated and stopped loading.

After the enrichment parameters of macroporous resin were optimized, a column (17 mm × 50 mm) was packed with 100 g macroporous adsorbent resin and washed with 300 ml 95% ethanol, 5% HCl, and 2% NaOH. After each wash, water was used to eliminate the residual 95% ethanol, HCl, or NaOH. 40 ml water extracts were then subjected to the column, eluting with water, 30% ethanol, 60% ethanol, and 95% ethanol to yield four fractions. The flow rate of extracts was 1.5 ml/min. Sixty percent of ethanol fraction was evaporated at 50°C under vacuum and freeze‐dried for later use.

### 2.6 HPLC conditions for analysis of the GAs and flavonoid contents of 60% ethanol fraction

HPLC conditions were determined according to the Methods of Determination of the GAs and Flavonoid Contents in Chinese Pharmacopeia 2015. Sixty percent ethanol fraction was examined on an Agilent Eclipse Plus C18 column (150 mm × 4.6 mm, 5 μm) operated at 30°C with the elution solvents *A* (0.1% TFA in acetonitrile) and *B* (0.1% TFA in water). Ginkgoneolic acid and total GAs were used to be the reference substance and positioning reference substance, respectively. Two gram of 60% ethanol fraction was dissolved in methanol and filtered through a 0.22 μm filter membrane before injection. The flow rate was set at 1 ml/min, and the detection wavelength was 310 nm. The injection volume was 50 μl. The gradient was as follows: 75% *B* at the beginning, 90% *B* to 30 min, 90% *B* to 35 min, 75% *B* to 36 min, and 75% *B* to 45 min.

Most of the flavonoids of *G. biloba* leaves are flavonoid glycosides. And, the main aglycone types of kaempferol, quercetin, and isorhamnetin could be obtained through hydrolysis with hydrochloric acid. Thus, the content of flavonoids could be calculated by determining the aglycones content and multiplying by a conversion factor (the conversion factors are different in US. Ph., Chin. Ph., and Eur. Ph., and an average value of 2.51 was used in Chin. Ph.; Liu et al., 2015b). The flavonoid content of 60% ethanol fraction was examined on an Agilent Eclipse Plus C18 column (150 mm × 4.6 mm, 5 μm) operated at 30°C with the elution solvents 0.4% phosphoric acid–water/methanol (1:1). Quercetin was used to be a reference substance. Thirty‐five milligram of the extract was dissolved in 25 ml mixture solution of methanol/25% hydrochloric acid (4:1). Heated and refluxed in a water bath for 30 min, cooled to room temperature immediately, then transferred to a 50 ml volumetric flask, diluted to scale mark, shake well, filtered, and the subsequent filtrate was then obtained. The flow rate was set at 1 ml/min, and the detection wavelength was 360 nm. The injection volume was 10 μl.

### 2.7 UPLC‐QTOF‐MS/MS conditions for structural identification of the flavonoids

The 60% ethanol fraction was analyzed through an Agilent series 1290 UPLC machine (Agilent) coupled with a QTOF instrument (Agilent). UPLC was equipped with an autosampler, a binary pump, a column heater, and a degasser. The sample was separated on a ZORBAX Eclipse Plus C18 (RRHD) column (50 mm × 2.1 mm, particle size, 1.8 μm). The mobile phase used was *A* (0.1% formic acid in water) and *B* (0.1% formic acid in acetonitrile). The gradient elution was performed as follows: 10%–20% *B* at 0–5 min, 20–25% *B* at 5–15 min, and 25%–35% *B* at 15–25 min. The flow rate was 0.2 ml/min at a column temperature of 30°C. The injection volume was 2 μl and the detection wavelength was 360 nm. The ESI‐QTOF spectra were acquired in negative mode, and the optimized parameters were as follows: primary scan range from *m*/*z* 100 to 1700, needle voltage at 3.5 kV, the capillary temperature at 350°C, nitrogen as the drying gas at 8 L/min, and nebulizer pressure at 35 psi.

### 2.8 Antioxidant assays

The antioxidant activities of water extracts after heat treatment, water extracts without heat treatment, and 60% ethanol fraction were evaluated by various antioxidant assays, including reducing power, DPPH radical scavenging, and OH^·^ scavenging. BHT was used as a standard antioxidant to compare them. UV–vis spectrophotometer of Agilent Cary 60 was used to test absorbance. The value error bar is SEM. All the activity assays were performed in three independent experiments.

#### 2.8.1 Reducing power assay

The reducing power of water extracts after heat treatment, water extracts without heat treatment, and 60% ethanol fraction was determined according to the method (Kumaran & Karunakaran, 2007) with a few modifications. Different amounts of water extracts after heat treatment (0.05–0.45 mg), water extracts without heat treatment (0.05–0.45 mg), and 60% ethanol fraction (0.025–0.35 mg) in 1 ml of methanol were mixed with phosphate buffer (2.0 ml and 0.2 mol/L, pH 6.6) and potassium ferricyanide [K_3_Fe(CN)_6_] (2.0 ml, 1%). The mixture was incubated at 50 °C for 20 min. A portion (2.0 ml) of trichloroacetic acid (10%) was added to the mixture, which was then centrifuged (4500 r/min at room temperature) for 10 min. The upper layer of solution (2.0 ml) was mixed with distilled water (1.0 ml) and FeCl_3_ (0.2 ml, 0.1%), and the absorbance was measured at 700 nm. Increased absorbance of the reaction mixture indicated increased reducing power.

#### 2.8.2 DPPH radical scavenging activity assay

The free radical scavenging activities of samples of water extracts after heat treatment, water extracts without heat treatment, and 60% ethanol fraction, based on the scavenging activity of the stable DPPH free radical, were determined by the method (Kumaran & Karunakaran, 2007). 0.1 ml of a methanolic solution of different concentrations was added to 3 ml of 0.004% MeOH solution of DPPH^·^. Absorbance at 517 nm was determined after 30 min, and the percent scavenging effect was calculated as Equation (5).

(5) $$\text{Scavenging rate}=\left[1-\left(A_{1}-A_{2}\right)/A_{0}\right]\times 100\%$$

where *A*_0_ was the absorbance of the control (without sample), *A*_1_ was the absorbance in the presence of the sample, and *A*_2_ was the absorbance without DPPH^·^.

#### 2.8.3 OH ^·^ scavenging assay

The abilities of samples of water extracts after heat treatment, water extract without heat treatment, and 60% ethanol fraction to scavenge hydrogen peroxide were determined according to the method (Wang et al., 2008) with a few modifications. OH radicals were generated from FeSO_4_ and H_2_O_2_, and detected by their ability to hydroxylate salicylate. The reaction mixture (3 ml) contained 1 ml FeSO_4_ (6 mM), 1 ml H_2_O_2_ (6 mM), 1 ml sodium salicylate (6 mM), and varying concentrations of 60% ethanol fraction and BHT. After incubation for 1 h at 37°C, the absorbance of the hydroxylated salicylate complex was measured at 510 nm. The percentage scavenging effect was calculated as Equation (6).

(6) $$\text{Scavenging rate}=\left[1-\left(A_{1}-A_{2}\right)/A_{0}\right]\times 100\%$$

where *A*_0_ is the absorbance of the control (without sample), *A*_1_ is the absorbance in the presence of sample, and *A*_2_ is the absorbance without sodium salicylate.

## 3 RESULTS AND DISCUSSION

### 3.1 Standard curve of rutin and *G. biloba* leaves bioactive flavonoid content of water extracts

**FIGURE 2:**
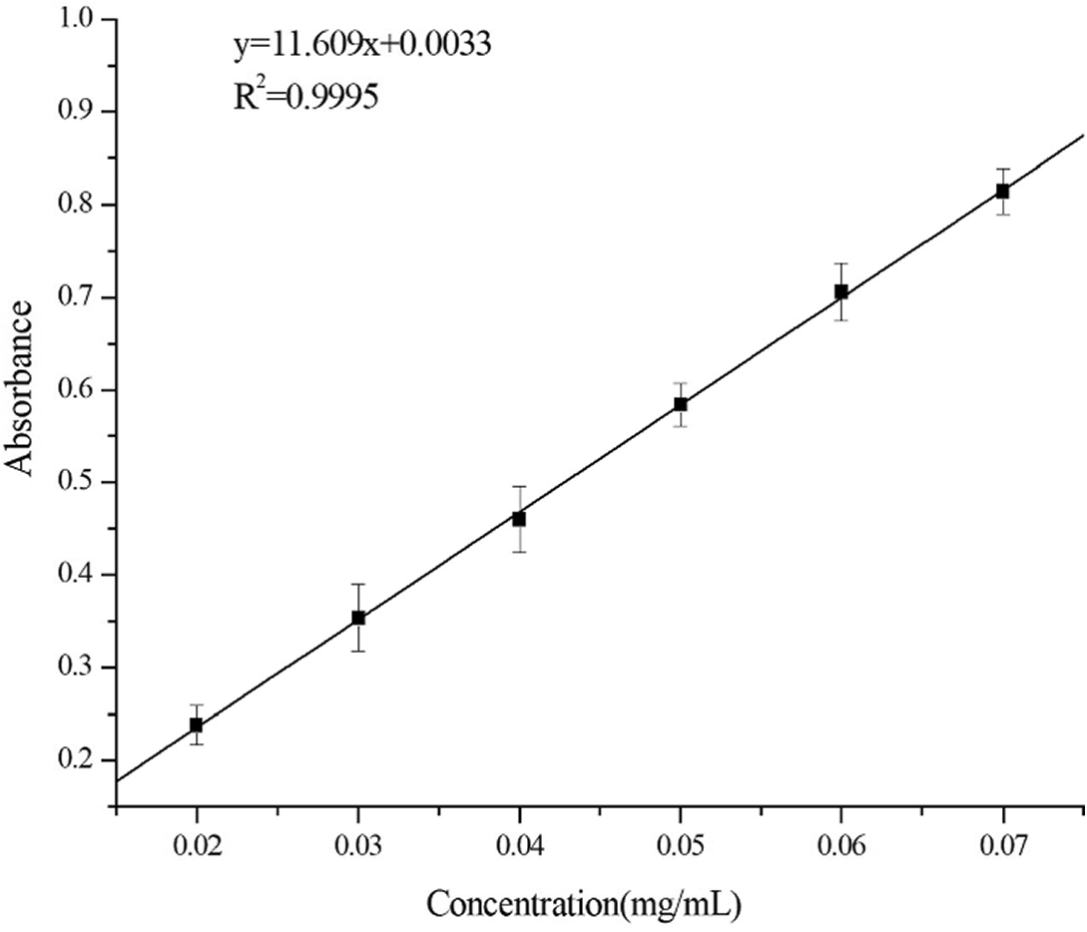
The rutin standard curve.

The rutin standard curve was drawn by plotting the concentration of the rutin standard solution versus the corresponding absorbency, as shown in Figure 2. The regression equation was Equation (7), where *A* was the absorbance of the sample, *C* (mg/ml) was the concentration of rutin, and *R* ^2^ of this equation was 0.9995.

(7) A=11.609C+0.0033

The GBLs flavonoid content of water extracts was calculated by Equation (1).

### 3.2 Factors influencing bioactive flavonoid content

There are many factors affecting the extraction yield of flavonoids. Three main factors of pretreatment temperature, extraction temperature, and liquid‐to‐solid ratio were investigated to separately study their influences. Single‐factor analysis was performed with one factor changed and the others kept unvaried.

**FIGURE 3:**
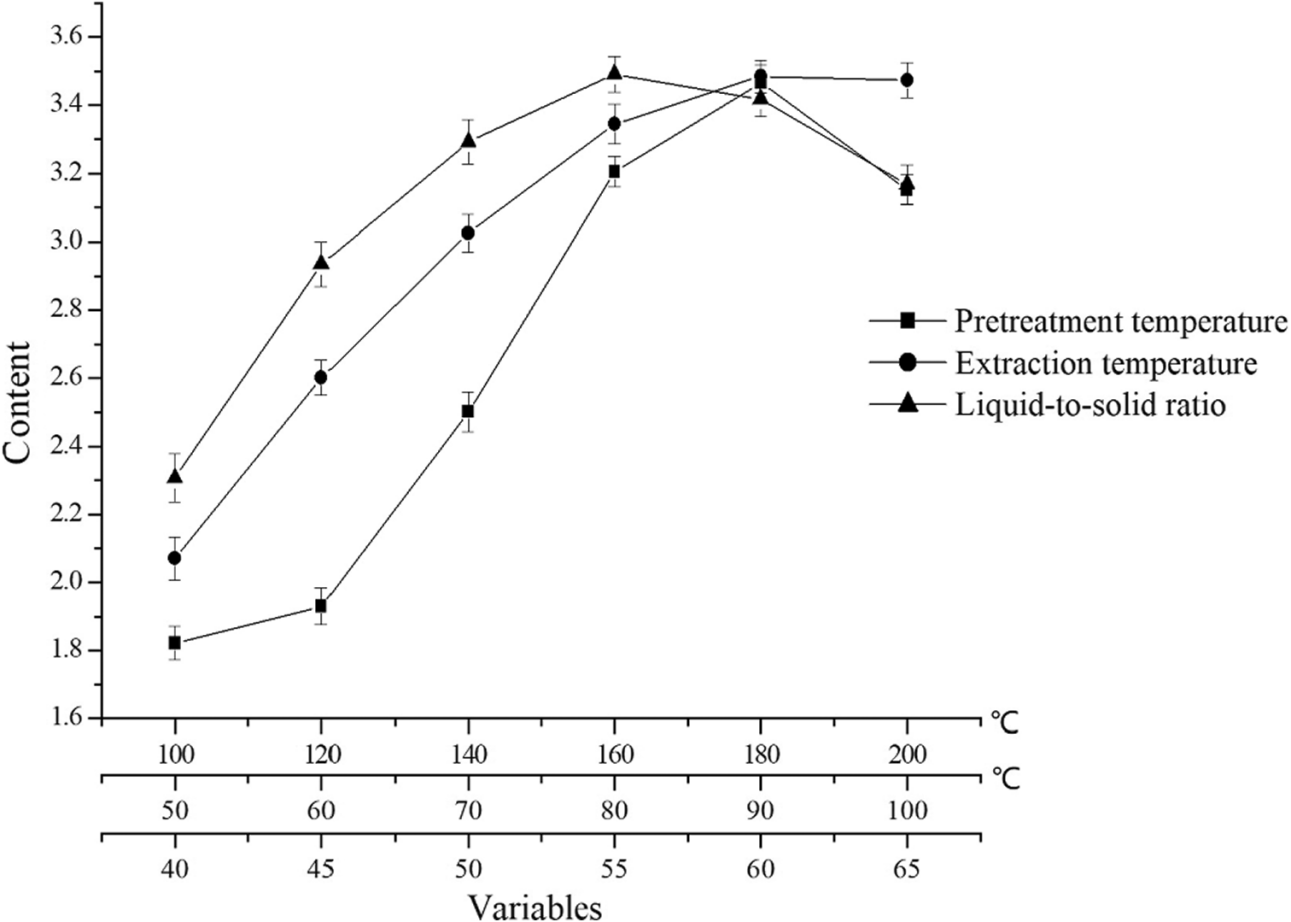
Experimental conditions and results of single‐factor analysis.

**FIGURE 4:**
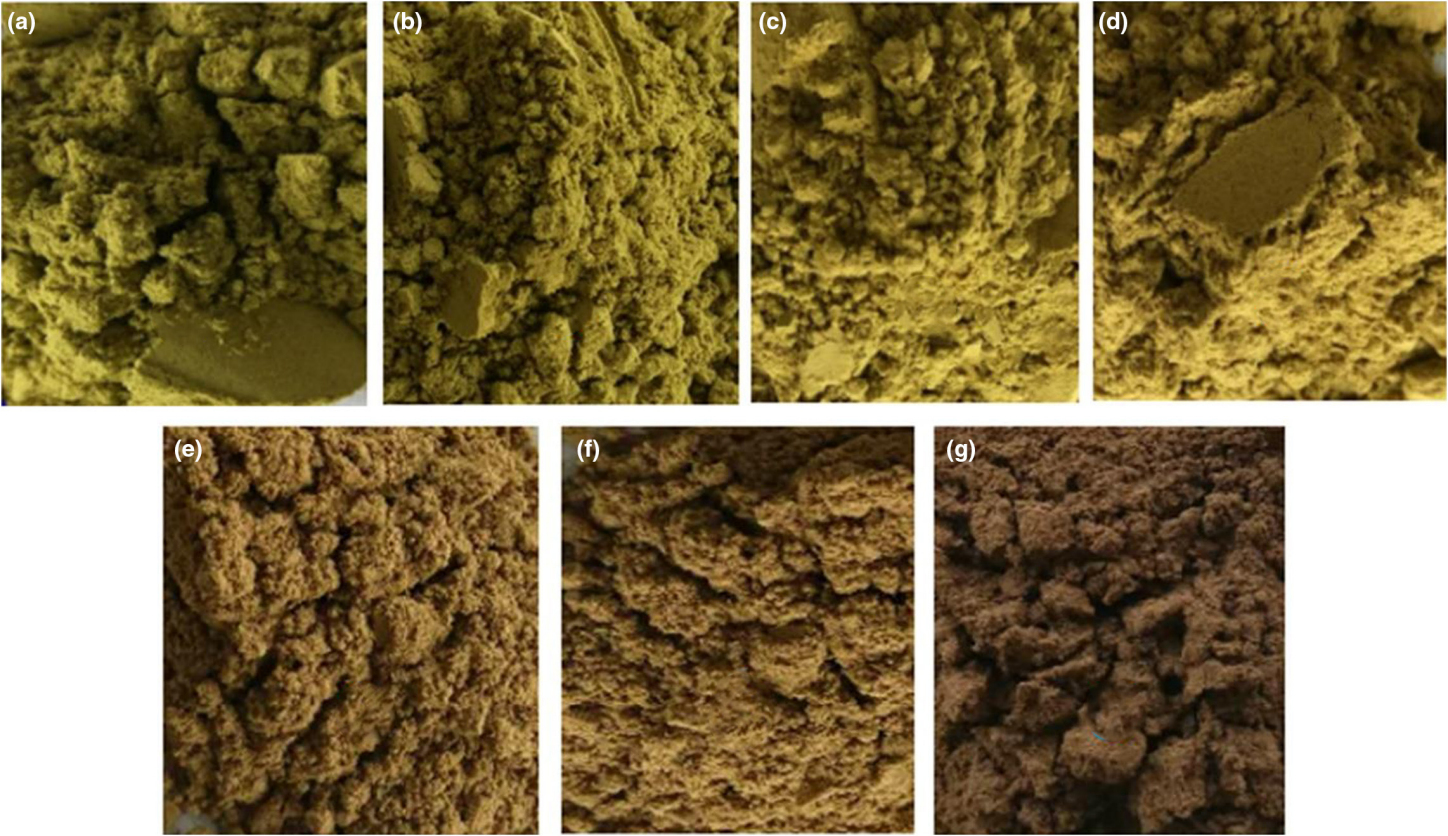
*Ginkgo biloba* leave powder with or without different high‐temperature pretreatment: (a) without high‐temperature pretreatment, (b) 100°C, (c) 120°C, (d) 140°C, (e) 160°C, (f) 180°C, and (g) 200°C.

The pretreatment temperature (100, 120, 140, 160, 180, and 200°C) was investigated, while the other conditions were kept the same (extraction temperature was 90°C, and liquid‐to‐solid ratio was 55:1). Extraction temperature (50, 60, 70, 80, 90, and 100°C) was investigated, while the other conditions were kept the same (pretreatment temperature was 180°C, and liquid‐to‐solid ratio was 55:1). Liquid‐to‐solid ratio (40:1, 45:1, 50:1, 55:1, 60:1, and 65:1) was investigated, while the other conditions were kept the same (pretreatment temperature was 180°C, and extraction temperature was 90°C). The detailed experimental conditions and results are shown in Figure 3. The content became higher with the pretreatment temperature increasing, and reached the highest when the pretreatment temperature was 180°C. And then, the content decreased instead. It could be speculated that some of the heat‐sensitive components in GBLs decomposed at a higher temperature. The content increased rapidly following the increase in extraction temperature and reached a peak at 90°C, then the content changed little with the temperature increase to 100°C. The content increased as the flavonoids dissolved more with the extraction temperature becoming higher. The variation trend of the liquid‐to‐solid ratio was similar to the pretreatment temperature, and reached a peak at 55:1. Although the flavonoids dissolved more with the increase in liquid‐to‐solid ratio, the increase in other components may reduce the content. Therefore, center point (all variables were coded as 0) of RSM was 180°C (pretreatment temperature) and 90°C (extraction temperature), 55:1 (liquid‐to‐solid ratio). As shown in Figure 4a–g, the colors of the GBLs changed from dark green to brown and then finally, to dark brown with the pretreatment temperature increases.

### 3.3 Optimization of experimental conditions by the RSM

BBD used in the present work was a three‐factorial design with three levels that consisted of 17 runs to obtain an optimal process condition. The experimental conditions and flavonoid content results are shown in Table 2. Additionally, an analysis of variance (ANOVA) was conducted and the regression model was summarized in Table 3. By multiple regression analysis, the following second‐order polynomial could be obtained as Equation (8).

(8) $$\begin{array}{c} Y=3.48-0.057A+0.066B+0.080C+0.076\mathrm{AB}-0.040\mathrm{AC}\\ \;-0.021\mathrm{BC}-0.25A^{2}-0.11B^{2}-0.070C^{2} \end{array}$$

**TABLE 2:** Response surface methodology design arrangement and the experimental results

Number	*A* (Pretreatment temperature)	*B* (Liquid‐to‐solid ratio)	*C* (Extraction temperature)	Content (%)
1	160	60	90	3.16902
2	200	55	100	3.12114
3	160	50	90	3.16902
4	180	55	90	3.47609
5	200	60	90	3.21945
6	180	55	90	3.45183
7	160	55	100	3.32606
8	200	50	90	2.91621
9	180	60	80	3.28010
10	160	55	80	3.11731
11	180	55	90	3.51631
12	180	50	100	3.35862
13	180	60	100	3.42757
14	180	50	80	3.12624
15	180	55	90	3.45055
16	200	55	80	3.07070
17	180	55	90	3.49716

**TABLE 3:** Analysis of variance (ANOVA)

Source	Sum of squares	df	Mean square	*F* value	*p*‐Value Prob > *F*	
Model	0.50	9	0.056	63.096	<.0001	Significant
*A*	0.026	1	0.026	29.05	.0010	
*B*	0.035	1	0.035	39.01	.0004	
*C*	0.051	1	0.051	57.58	.0001	
*AB*	0.023	1	0.023	25.93	.0014	
*AC*	0.006	1	0.006	7.07	.0325	
*BC*	0.002	1	0.002	2.03	.1970	
*A* ^2^	0.262	1	0.262	295.98	<.0001	
*B* ^2^	0.051	1	0.051	57.79	.0001	
*C* ^2^	0.021	1	0.021	23.23	.0019	
Residual	0.006	7	0.001			
Lack of fit	0.00	3	0.00	1.19	.4186	Not significant
Pure error	0.00	4	0.00			
Cor total	0.510	16				

As can be seen in Table 3, *F* value of the model was 63.096 while the *p* value was <.0001, which showed a high significance of the model. For a good accuracy of a model, *R* ^2^ must be more than 75% (Chauhan & Gupta, 2004). Thus, the model stated above with a relatively high coefficient of determination value (*R* ^2^ = 0.9878) illustrated that almost all extraction data could be explained by this model. Therefore, using this model to predict the influence of the process variables on the flavonoid content was reasonable and reliable. The linear variables *B* and *C* and quadratic variables *A* ^2^ and *B* ^2^ were statistically very significant because the *p* value was .0004, .0001, <.0001, and .0001, respectively (*p* < 0.001); linear variable *A*, two‐variable interaction *AB*, and quadratic variable *C* ^2^ had significant influences on the yield of flavonoids for the *p* value was .001, .0014, and .0019, respectively (*p* < .01); two‐variable interaction *AC* had influences for the *p* value of .0325 (*p* < .05). The linear and quadratic coefficients of each process variable indicated that the influence of factors was extraction temperature > liquid‐to‐solid ratio > pretreatment temperature.

**FIGURE 5:**
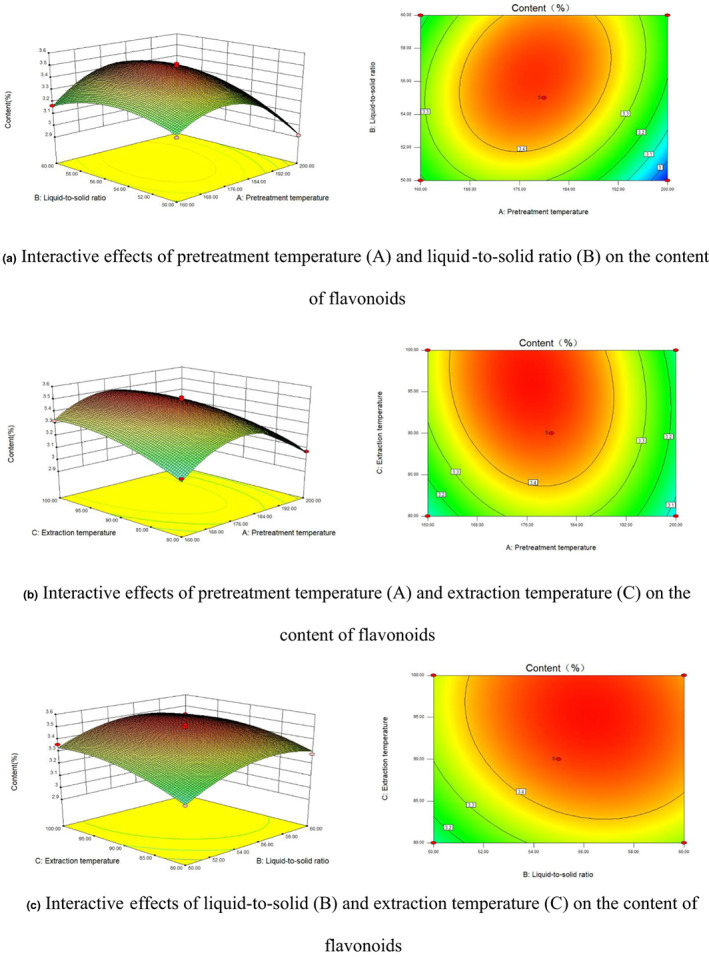
Interactive effects of pretreatment temperature, liquid‐to‐solid ratio, and extraction temperature on the content of *G. biloba* leaves flavonoids (as predicted by 3D surfaces and contour plots).

The effects of the process variables and their mutual interactions on the content can be investigated by the response surface plots and their contour plots. And the interactions between the process variables are significant while the shape of contour plots is elliptical. The 3D surfaces and contours plotted in Figure 5a–c displays the effect of extraction temperature on flavonoid content showing the most obvious in all conditions.

According to the analysis of the response surface, the optimum condition was obtained: pretreatment temperature, 177.4°C; liquid‐to‐solid ratio, 56:1 (ml/g); and extraction temperature, 95.8°C. Under the above condition, the estimated value for content was obtained, which was 3.51%. Considering the practical operability, the actual experiment condition was adjusted to be: pretreatment temperature, 177°C; liquid‐to‐solid ratio, 56:1 (ml/g); and extraction temperature, 96°C. After three parallel experiments, the mean value of flavonoid content was 3.38 ± 0.19%, which was close to the estimated value. By comparing the actual experimental and predicted content, the model had a great agreement with the predictive values and could be verified. Therefore, the optimal extraction conditions obtained by RSM were accurate, reliable, and efficient.

### 3.4 Determination of optimized enrichment parameters of macroporous resin

Adsorbing capacity and desorption rate were important indicators to evaluate resin. A higher adsorption rate and desorption rate could recover effective constituents to the maximum extent. The static adsorption and desorption characteristics of the three macroporous resins are shown in Table 4. After overall consideration of adsorbing capacity, adsorption rate, and desorption rate, D380 was selected to further purify the water extracts.

**TABLE 4:** The static adsorption and desorption characteristics of the three macroporous resins

Type	Adsorbing capacity (mg/g)	Adsorption rate (%)	Desorption rate (%)
AB‐8	14.58	70.37	54.90
D380	15.13	73.05	55.32
DA201	14.23	68.67	58.54

### 3.5 Contents of bioactive flavonoids and toxic GAs of water extracts and 60% ethanol fraction, and content changes in toxic GAs after pretreatment

**FIGURE 6:**
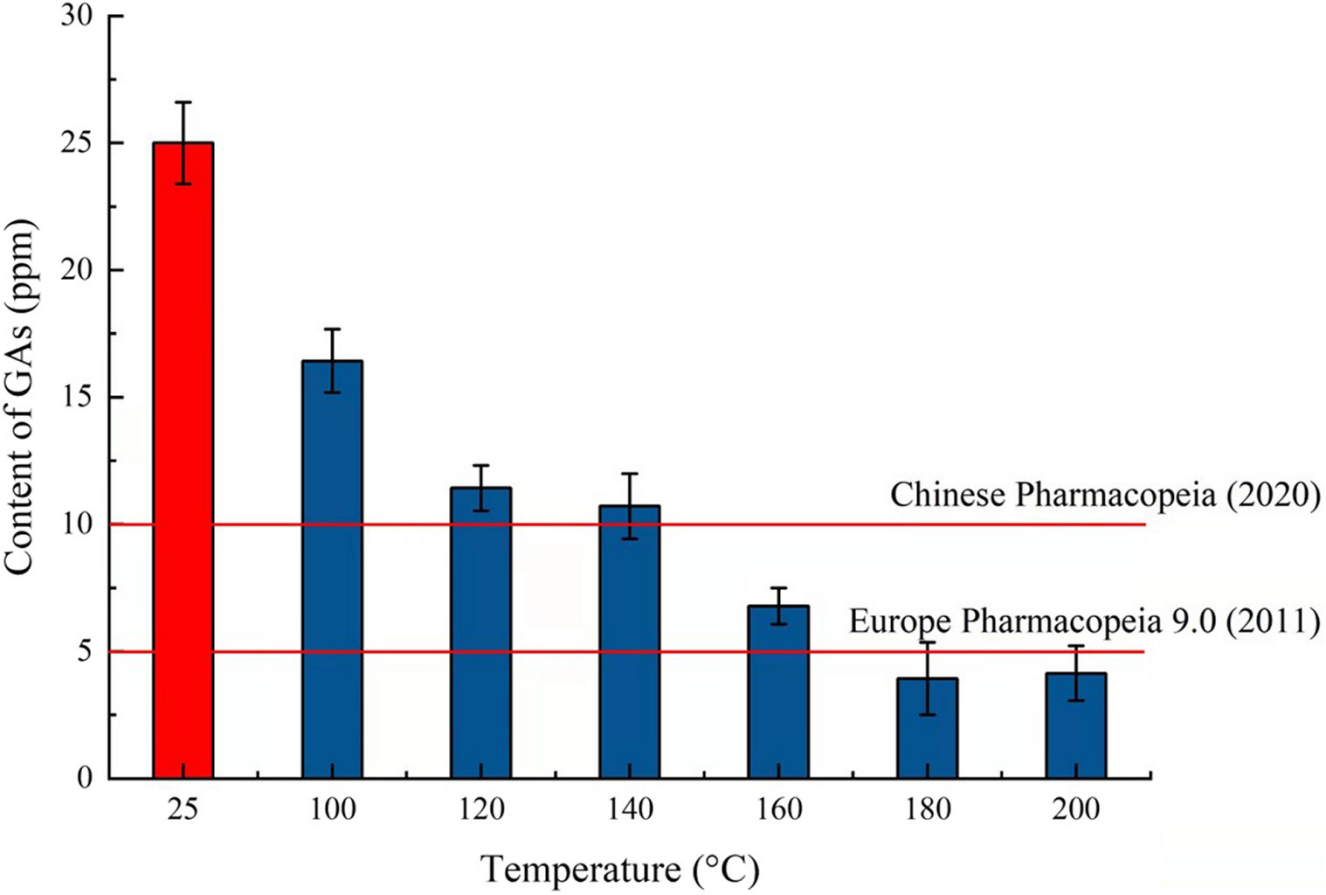
The content changes in toxic constituents of GAs in *Ginkgo biloba* leaves before and after high‐temperature pretreatment.

Finally, the flavonoid content by the conventional method was also conducted using the above optimum condition, and the new process with high‐temperature pretreatment increased by about 44.30% compared with the traditional water extraction method. The contents of GAs using ethanol and water as extraction solvents without high‐temperature pretreatment were firstly determined. The content of GAs was 20640.85 ppm after ethanol extraction for three times, and the content of GAs was 126.31 ppm after water extraction for three times, so only using water extraction could not reduce the toxic GAs to the limit of the requirements of Chinese Pharmacopeia (2020) and Europe Pharmacopeia 9.0 (2011), and the subsequent processing of removing GAs was still needed. The content changes in toxic constituents of GAs in *Ginkgo biloba* leaves after high‐temperature pretreatment are shown in Figure 6. The content of GAs using the traditional water extraction method was 24.91 ppm for one time. However, compared with the traditional method, the content of toxic constituents of GAs decreased significantly with the pretreatment temperature increase from 100°C to 200°C (*p* < .05). When the pretreatment temperature reached 160°C, the content of GAs could reduce 6.73 ppm and fit the requirements of Chinese Pharmacopeia (2020; <10 ppm), and when the pretreatment temperature reached 180°C and 200°C, the content of GAs could reduce to 4.11 ppm and 4.07 ppm and fit the requirements of Europe Pharmacopeia (2011; <5 ppm). Thus, proper temperature pretreatment could dramatically reduce the content of toxic GAs and high‐temperature pretreatment really helps to significantly reduce the content of GAs.

After purification with macroporous resin, the content of bioactive flavonoids increased to 43.50% while the GAs were not detected. Thus, the subsequent complicated and tedious work of purification to remove GAs could be omitted, which could further reduce the cost of production.

### 3.6 Identification of flavonoids by UPLC‐QTOF‐MS/MS

**FIGURE 7:**
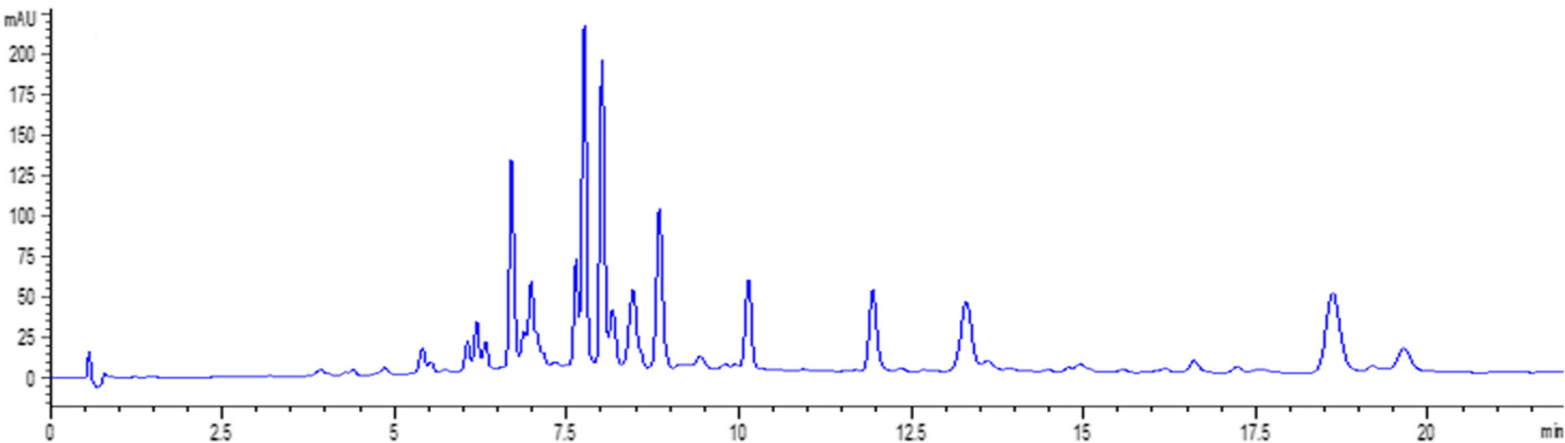
UV chromatogram at 360 nm of the 60% ethanol extracts.

**FIGURE 8:**
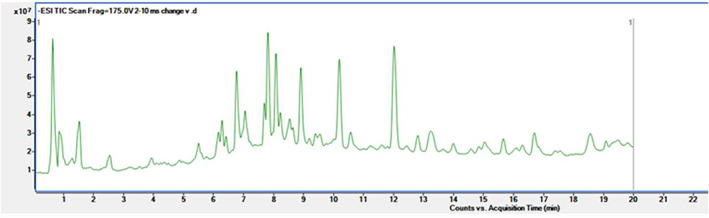
The total ion current chromatogram of the 60% ethanol extracts.

The 60% ethanol fraction was detected using UPLC‐QTOF‐MS/MS. The UV chromatogram at 360 nm is shown in Figure 7 and the UPLC‐QTOF‐MS/MS total ion chromatogram is shown in Figure 8. Compounds **1**–**11** were identified by comparing their MS spectra with reference literature (Li et al., 2020; Sun et al., 2015; Wang, 2007), and compounds **12**–**14** were compared with standards. The comprehensive profiling of phytochemicals of compounds was summarized in Table 5. The mass spectrogram of each compound was shown in Figures S1–S14. As shown in Figures 7 and 8, Table 5, and Figures S1–S14, quercetin, kaempferol, and isorhamnetin glycosides were found. Of all the identified 14 compounds, compounds **1**–**11** were all flavonoid glycosides and compounds **12**–**14** were aglycones, so the flavonoid glycosides were not decomposed by the high temperature.

**TABLE 5:** List of compounds of 60% ethanol fraction.

Id#	Compound	*m*/*z* MS	Adduct	RT	*m*/*z* MS/MS	Reference
**1**	3‐O‐[6‐O‐(α‐l‐rhamnosyl)‐β‐d‐glucosyl] myricetin	625.1399	M–H	5.570	316, 271	Chauhan and Gupta (2004)
**2**	3‐O‐[2‐O‐(6‐O‐*p*‐coumaroyl)‐β‐d‐glucosyl]‐α‐l‐rhamnosyl‐7‐O‐(β‐d‐glucosyl) quercetin	917.2349	M–H	6.118	755, 609, 462, 300	Chauhan and Gupta (2004)
**3**	3‐O‐[2‐O, 6‐O‐bis (α‐l‐rhamnosyl)‐β‐d‐glucosyl] kaempferol	739.2077	M–H	6.243	284	Chauhan and Gupta (2004)
**4**	3‐O‐[2‐O, 6‐O‐bis (α‐l‐rhamnosyl)‐β‐d‐glucosyl] isorhamnetin	769.2178	M–H	6.360	314	Chauhan and Gupta (2004)
**5**	3‐O‐[6‐O‐ (α‐l‐rhamnosyl)‐β‐d‐glucosyl] quercetin	609.1441	M–H	6.810	464, 300	Chauhan and Gupta (2004)
**6**	3‐O‐[6‐O‐ (α‐l‐rhamnosyl)‐β‐d‐glucosyl]‐3‐methylmyricetin	639.1552	M–H	7.077	331	Chauhan and Gupta (2004)
**7**	3‐O‐(β‐d‐glucosyl] quercetin	463.0000	M–H	7.162	301	Chauhan and Gupta (2004)
**8**	3‐O‐[6‐O‐(α‐l‐rhamnosyl)‐β‐d‐glucosyl] kaempferol	593.1490	M–H	7.823	285	Chauhan and Gupta (2004)
**9**	Kaempferol 3‐O‐β‐d‐glucoside	447.0851	M–H	8.162	284, 255	Wang (2007)
**10**	7‐O‐β‐d‐glucosyl apigenin	431.0948	M–H	8.529	268	Chauhan and Gupta (2004)
**11**	Quercetin 3‐O‐[2‐O, 6‐O‐bis (α‐l‐rhamnosyl)‐β‐d‐glucoside]	755.1827	M–H	10.221	609, 300	Li et al. (2020)
**12**	Quercetin	301.0337	M–H	13.293	151, 107	Chauhan and Gupta (2004)
**13**	Kaempferol	285.0383	M–H	18.509	227, 159, 117	Chauhan and Gupta (2004)
**14**	Isorhamnetin	315.0000	M–H	19.447	300, 271, 151	Chauhan and Gupta (2004)

### 3.7 Antioxidant activity

#### 3.7.1 Reducing power assay

**FIGURE 9:**
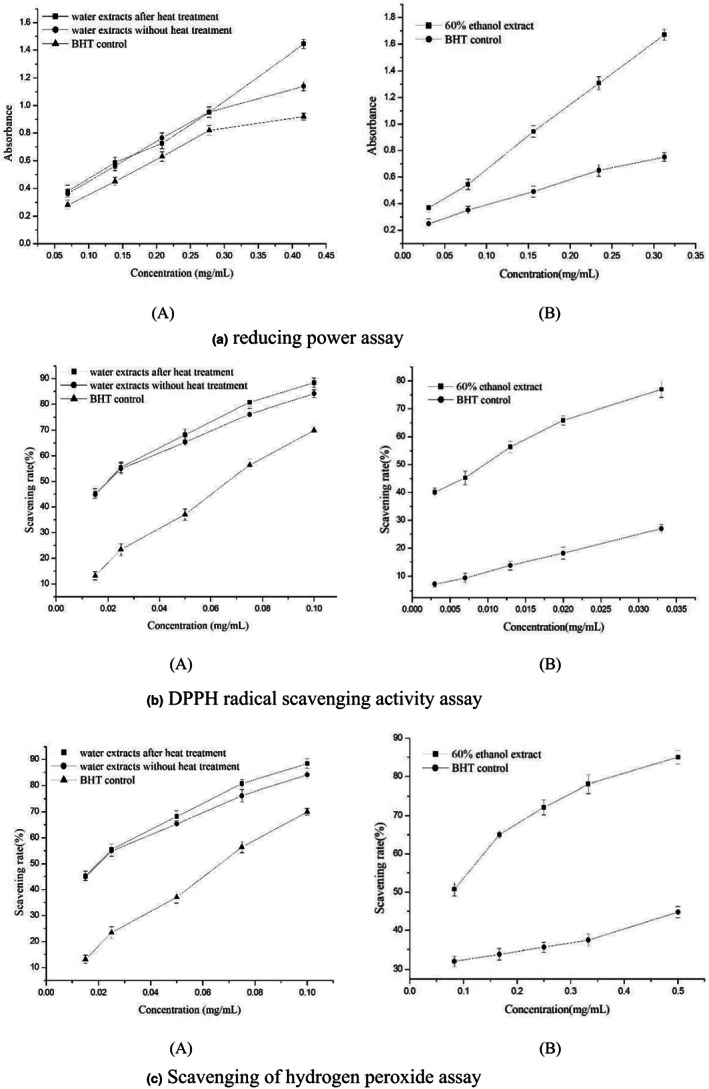
The antioxidant activities of water extracts after heat treatment, water extracts without heat treatment, and 60% ethanol fraction. The absorbance values were directly plotted as the mean of replicate absorbance values ±1 SD (*n* = 3) against extract concentration in mg extract per ml reaction volume. The absorbance values were converted to scavenging rate (%) and data plotted as the means of replicate scavenging rate (%) values ± 1 SD (*n* = 3) against extract concentration in mg extract per ml reaction volume.

The reducing properties are generally associated with the presence of reductones (Duh, 1998). An electron‐donating reducing agent contributes to antioxidant activity by its capacity to donate an electron to free radicals, which results in the neutralization of the radical, and the reduced species subsequently acquire a proton from the solution. Another reaction pathway in electron donation is the reduction of an oxidized antioxidant molecule to regenerate the “active” reduced antioxidant (Li et al., 2020). BHT, with its strong ability to scavenge free radical, was used as the standard to evaluate the free radical scavenging activity of extracts. Figure 9a(A) shows the reductive capability of water extracts used in the new method compared to conventional method. The reducing power of water extracts of *G. biloba* leaves used in the two methods both increased and had similar tendencies at the same dose and were all better than BHT. Figure 9a(B) shows the reductive capability of 60% ethanol fraction compared to BHT. We used absorbance values to characterize the reducing powder activity of 60% ethanol fraction. The reducing power of 60% ethanol fraction of *G. biloba* leaves increased with an increasing amount of sample and showed stronger reducing powder activity than BHT at the same dose. Sixty percent ethanol fraction had an influence on the reducing power when the dose was 0.156 mg/ml (*p <* .05) and had a significant influence when the dose was 0.234 mg/ml and 0.312 mg/ml (*p* < .01). After purification, the activities of the 60% ethanol faction were significantly improved. And, compared with water extracts, 60% ethanol faction had better reducing power at a lower concentration.

#### 3.7.2 DPPH radical scavenging activity assay

The role of an antioxidant is to remove free radicals. As a stable and well‐characterized solid radical source, DPPH^·^ is a traditional and perhaps the most popular free radical used for free radical scavenging activity assay. BHT was used as the standard to evaluate the free radical scavenging activity of extracts. The absorbance values were converted to scavenging effects (%) to characterize the free radical scavenging activity of the 60% ethanol fraction. As shown in Figure 9b(A), the scavenging effects of water extracts used in the new method and conventional method both increased and had similar tendencies at the same dose and were all much better than BHT. As shown in Figure 9b(B), the scavenging effects of the 60% ethanol fraction and BHT both increased with the concentration added. However, 60% ethanol fraction showed a very significant, stronger DPPH^·^ scavenging activity than BHT at the same dose and had a very significant influence when the dose was 0.003, 0.007, 0.013, 0.02, and 0.033 mg/ml (*p* < .001). After purification, the activities of the 60% ethanol faction were significantly improved. And, compared with water extracts, 60% ethanol faction had better DPPH^·^ scavenging activity.

#### 3.7.3 OH ^·^ scavenging activity assay

Scavenging of OH^·^ is an important antioxidant activity because of the very high reactivity of the OH radical which enables it to react with a wide range of molecules found in living cells, such as sugars, amino acids, lipids, and nucleotides. Although OH^·^ formation can occur in several ways, by far the most important mechanism in vivo is the Fenton reaction, where a transition metal is involved as a pro‐oxidant in the catalyzed decomposition of superoxide and hydrogen peroxide (Wang et al., 2008). The absorbance values were converted to scavenging effects (%) to characterize the scavenging of OH^·^ activity of 60% ethanol fraction. BHT was used as the standard. As shown in Figure 9c(A), the scavenging effects of water extracts used in the new method and conventional method both increased with the concentration added and had similar tendencies and were all much better than BHT. As shown in Figure 9c(B), the scavenging effects of the 60% ethanol fraction and BHT both increased with increasing amount. However, 60% ethanol fraction showed a significantly stronger OH^·^ scavenging activity than BHT at the same dose and had a significant influence when the dose was 0.083 mg/ml (*p* < .01), and a very significant influence when the dose was 0.167, 0.25, 0.333, and 0.5 mg/ml (*p* < .001). After purification, the activities of the 60% ethanol faction were significantly improved. And, comparing with water extracts, 60% ethanol faction had better OH^·^ scavenging activity.

## 4 CONCLUSIONS

In the present study, high‐temperature pretreatment was developed to remove the main toxic constituents of GAs from GBLs and improve the content of bioactive flavonoids by water extraction. And, RSM in combination with three‐factor and three‐level BBD was successfully applied to study and optimize the process variables for the water extraction of flavonoids from GBLs. The experiment results showed that pretreatment temperature, extraction temperature, and liquid‐to‐solid ratio all had significant effects on the content. Analysis of variance (ANOVA) showed a high coefficient of determination value (*R* ^2^ > .95). Therefore, the mathematical model developed by BBD can be used to predict the extraction efficiency of flavonoids. Under the optimal conditions (pretreatment temperature: 177°C, extraction temperature: 96°C, and liquid‐to‐solid ratio: 56:1), the highest content of flavonoids could reach 3.38 ± 0.19%, which was in agreement with the predicted one. At the same time, the content of GAs could be reduced to 4.11 ppm, which is in accord with the requirements of Chinese Pharmacopeia (2015) and Europe Pharmacopeia 9.0 (2011). The content of toxic GAs substantially decreased by 83.50% while the content of bioactive flavonoids increased by 44.30% compared with the traditional water extraction method. After being purified by macroporous adsorbent resin, the content of bioactive flavonoids of 60% ethanol fraction increased to 43.50% while the GAs were not detected. Thus, compared with conventional methods, the new process was more efficient, environmentally friendly, and could also avoid subsequent processes of removing toxic GAs.

Sixty percent ethanol fraction was further analyzed by UPLC‐QTOF‐MS/MS and quercetin, kaempferol, and isorhamnetin glycosides were found. In addition, antioxidant activities including reducing power, DPPH radical scavenging, and OH^·^ scavenging assays all showed that the 60% ethanol fraction had much stronger antioxidant abilities than BHT.

## 5 DISCUSSION

Nowadays, the techniques of new “green and innovative” in food processing are ultrasound‐assisted processing, microwave processing, extrusion, supercritical fluid extraction and processing, controlled pressure drop process, pulse electromagnetic field, subcritical water extraction, high pressure, and so on. Food technology is currently a rapidly evolving field of applied research and industry under extreme or nonclassical conditions (Singla & Sit, 2021). The high‐temperature pretreatment process used in this article is economical and easy to operate. And, most important of all, the content of toxic constituents of GAs in *G. biloba* leaves decreased very significantly after pretreatment. The new method could reduce the content of toxic GAs to the limit of Chinese Pharmacopeia (2020) and Europe Pharmacopeia 9.0 (2011), so the subsequent processing to remove GAs was not needed, which will greatly reduce production costs and environmental pollution. Meanwhile, the content of flavonoids was greatly improved and the antioxidant activities were not affected using the new method.

Moreover, in the Chinese traditional tea industry, *G. biloba* leaf tea was usually pretreated by stir frying at a certain temperature to make it safe to drink. That is, the toxic components of *G. biloba* leaf tea could be removed after stir frying, which is coincided with the method of high‐temperature pretreatment used in this study. Thus, our study could lay a solid scientific basis on this traditional process. Furthermore, this high‐temperature, pretreated GBLs could also be used as materials to increase the yield in other food industries such as fermentation (Wang et al., 2013) or food additive for the obvious antioxidant activities.

As for standard *Ginkgo biloba* leaf extract, the content of ginkgo terpene lactones was also paid attention to. The literature showed that terpene lactones would decompose in the temperature range 280–430°C (Lü et al., 2011). In the study, the highest pretreatment temperature used was 200°C, so heat treatment will not affect the content of terpene lactones. Our experiment was a new attempt, and there is still a long way to go before it becomes a product. In this study, we focused on reducing GAs and improving the content of flavonoids. However, in order to evaluate a new method, there is still a lot of work to do in future studies. For example, the content and heat stability of terpene lactones, analyzing and comparing the structure and antioxidant activities of the 60% ethanol extract of Ginkgo leaves without heat treatment, and the 60% ethanol extract of Ginkgo leaves after heat treatment, and so on.

## CONFLICT OF INTEREST

The authors declare that there are no conflicts of interest.

## Supporting information


Figure S1

## Data Availability

The data that support the findings of this study are available in the Supporting Information of this article.
