# Supplementary material for: Reducing toxic constituents of ginkgolic acid content and improving bioactive flavonoid content from Ginkgo biloba leaves by high‐temperature pretreatment processing
Source: Food Sci Nutr. 2022 Oct 30;11(2):838–52. doi: 10.1002/fsn3.3118 (PMC9922130; doi:10.1002/fsn3.3118)
Supplement: Supplementary file 1 — Figure S1 [file FSN3-11-838-s001.docx]

**Supplementary Materials**

**
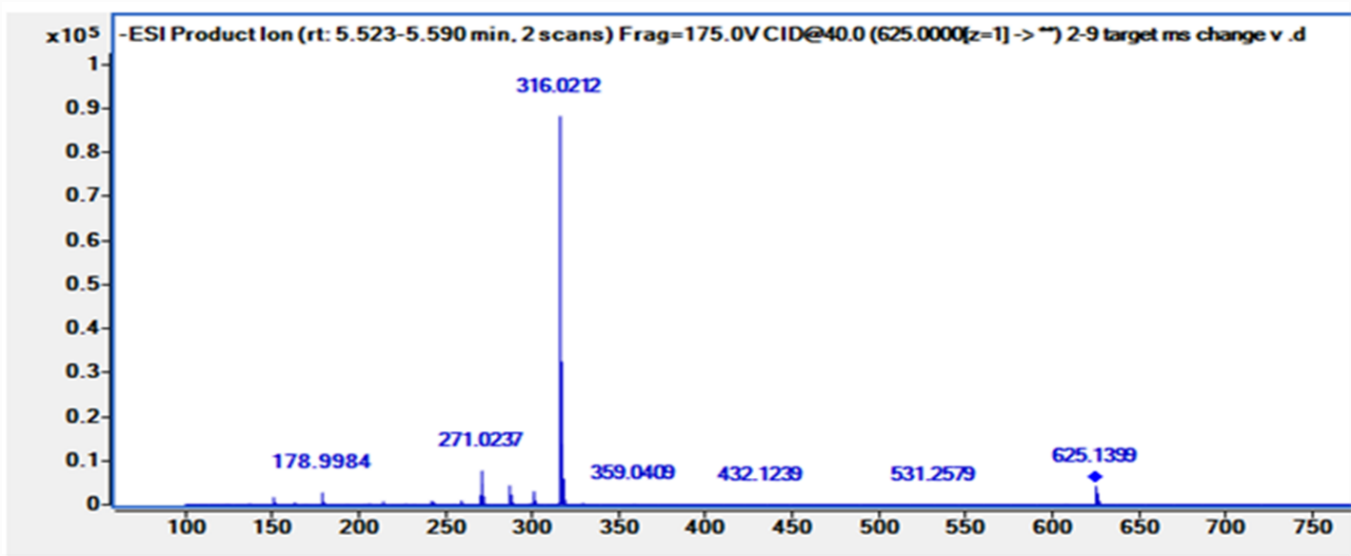
**

Figure S1: The mass spectrogram of Compound 1

**
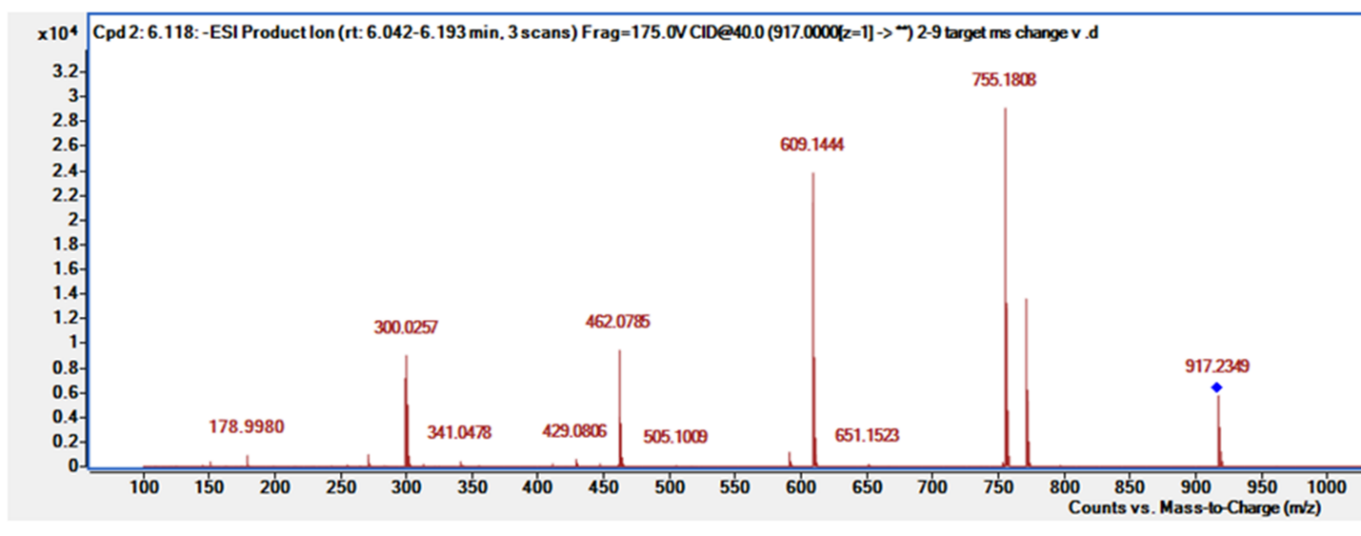
**

Figure S2: The mass spectrogram of Compound 2

**
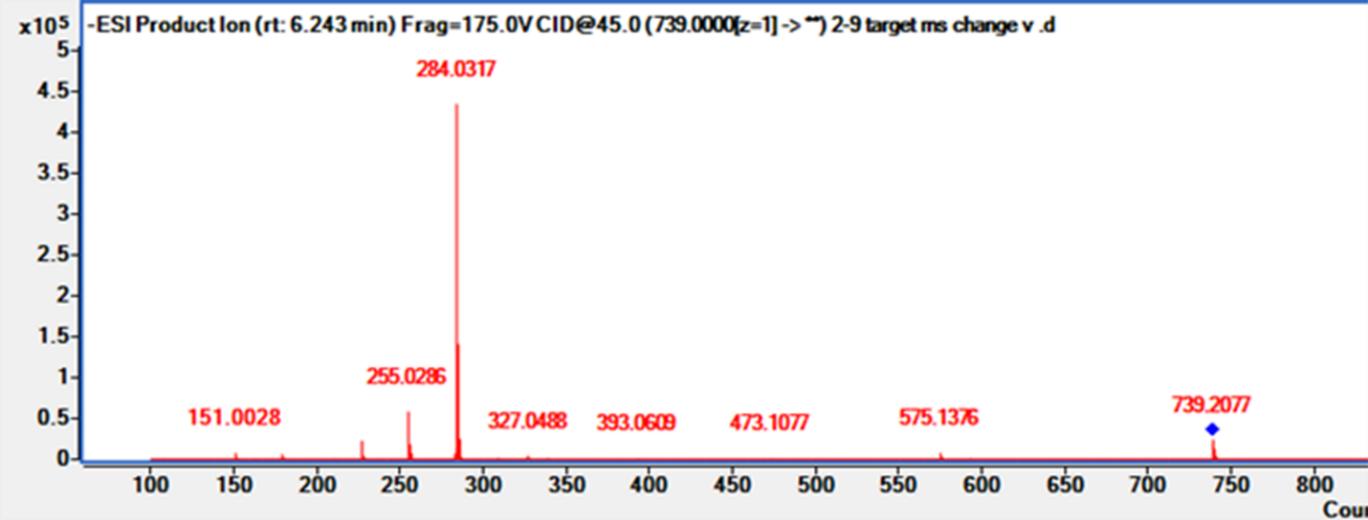
**

Figure S3: The mass spectrogram of Compound 3

**
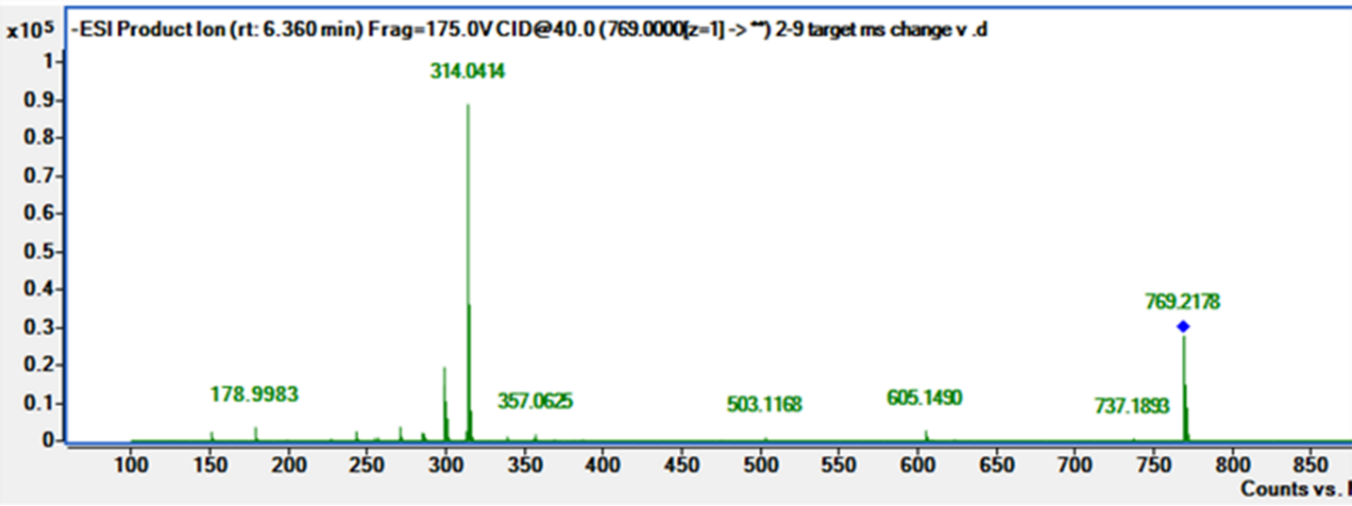
**

Figure S4: The mass spectrogram of Compound 4

**
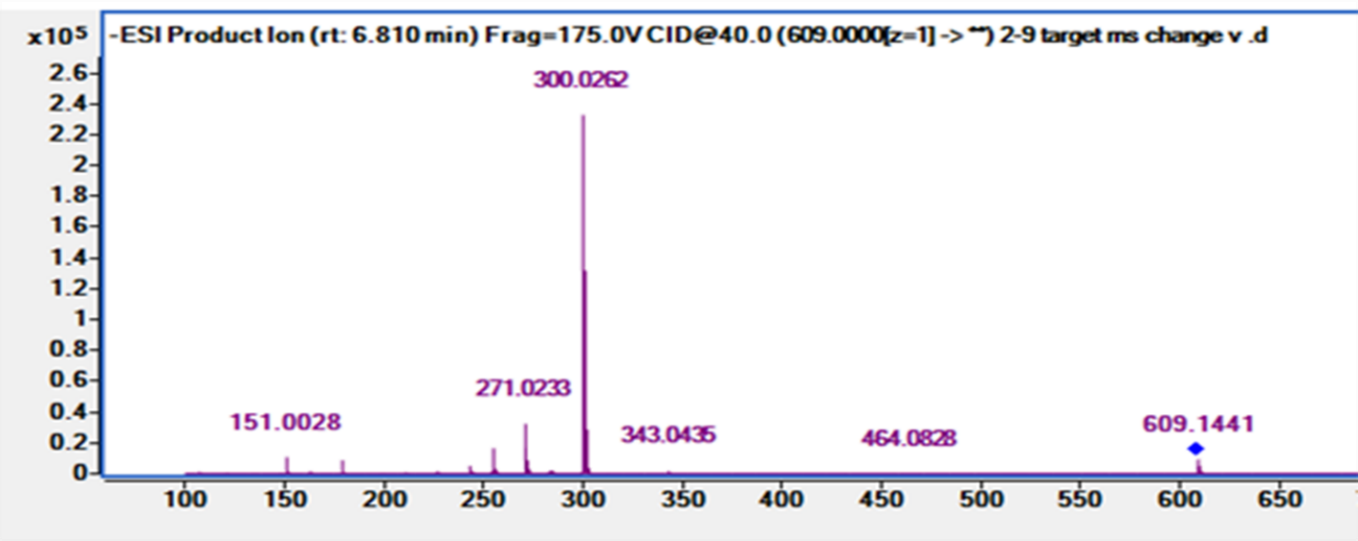
**

Figure S5: The mass spectrogram of Compound 5

**
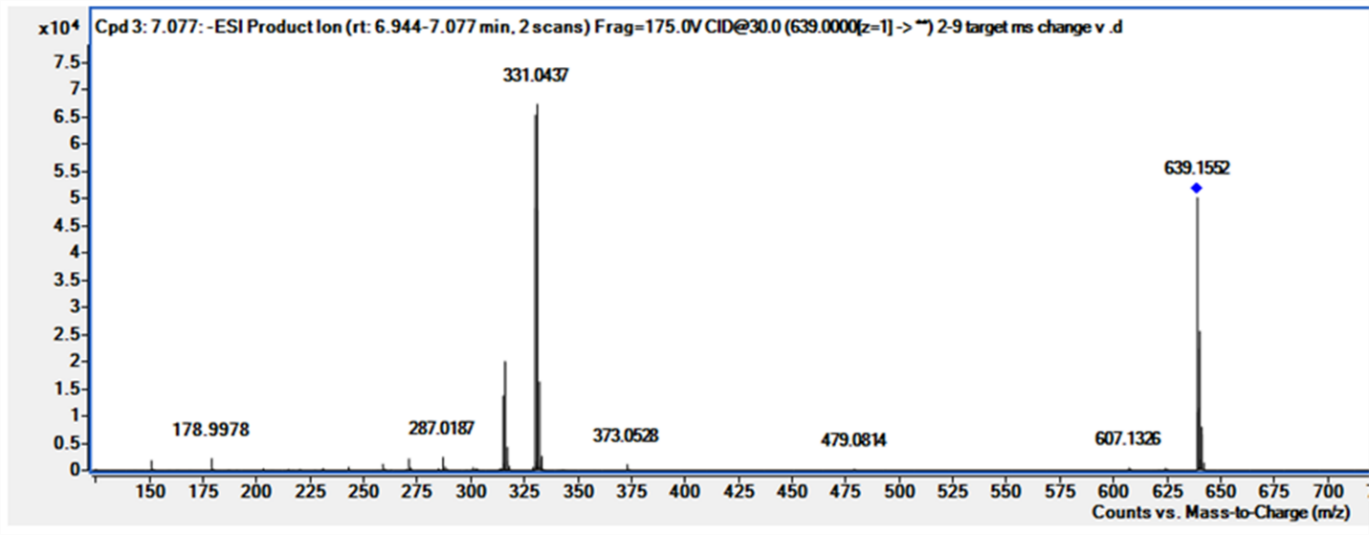
**

Figure S6: The mass spectrogram of Compound 6

**
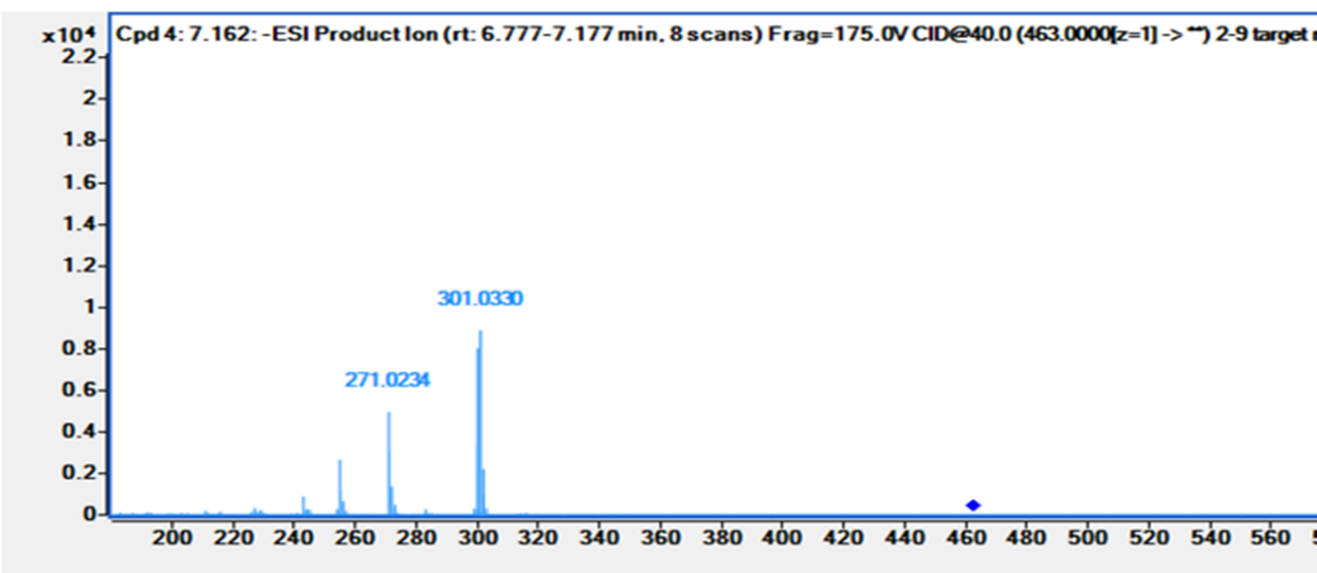
**

Figure S7: The mass spectrogram of Compound 7

**
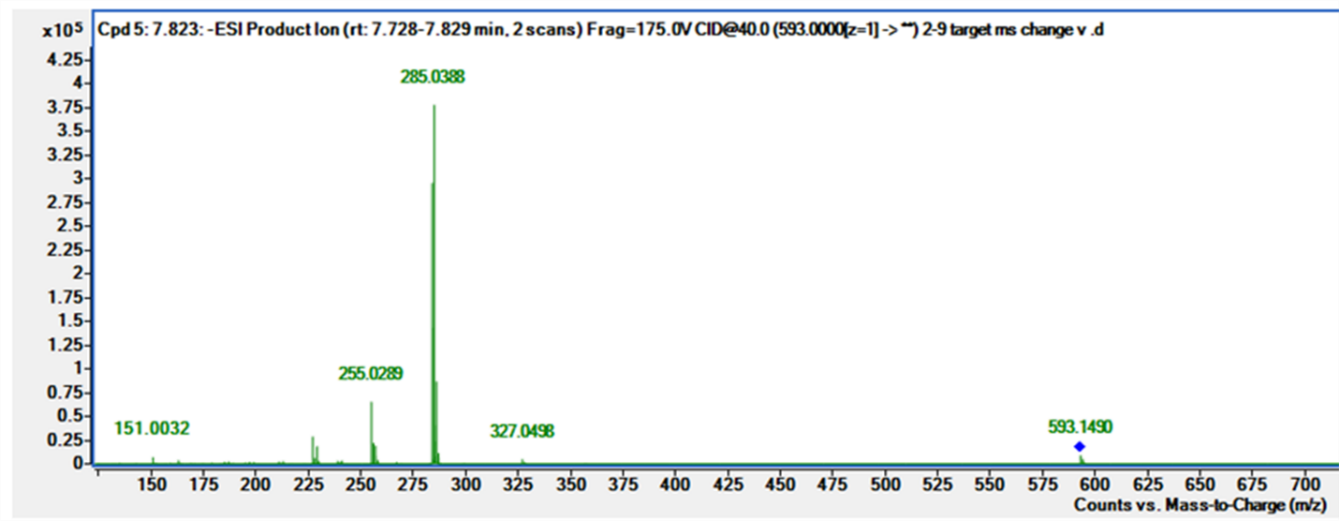
**

Figure S8: The mass spectrogram of Compound 8

**
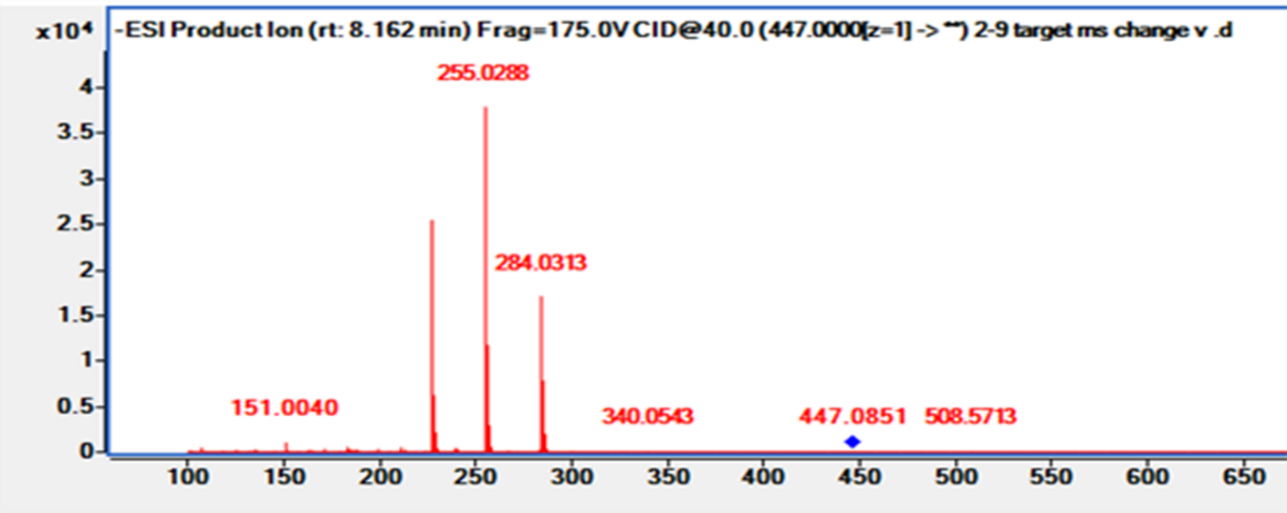
**

Figure S9: The mass spectrogram of Compound 9

**
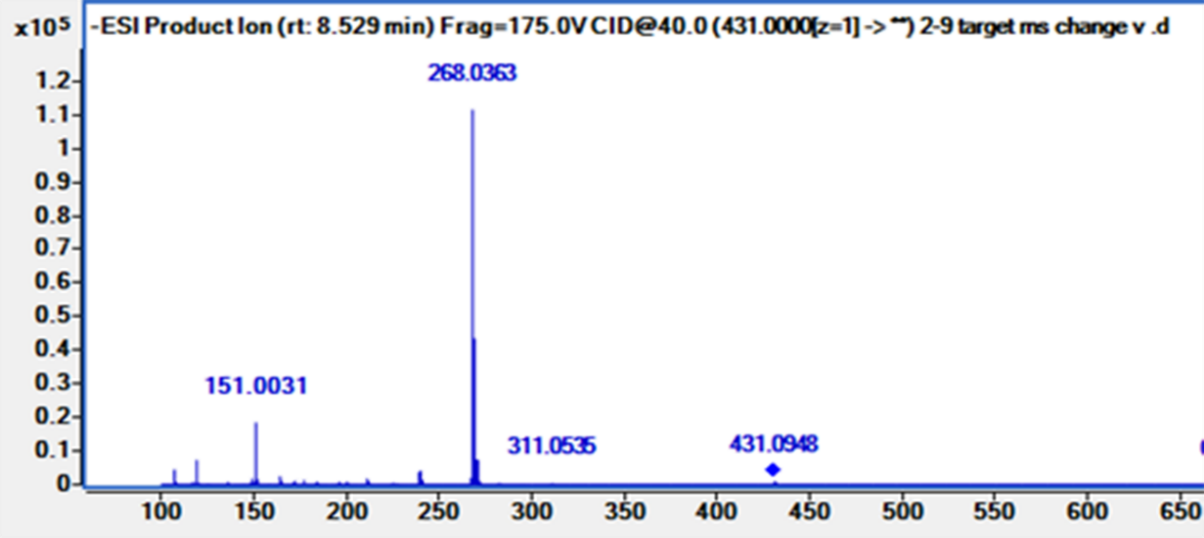
**

Figure S10: The mass spectrogram of Compound 10

**
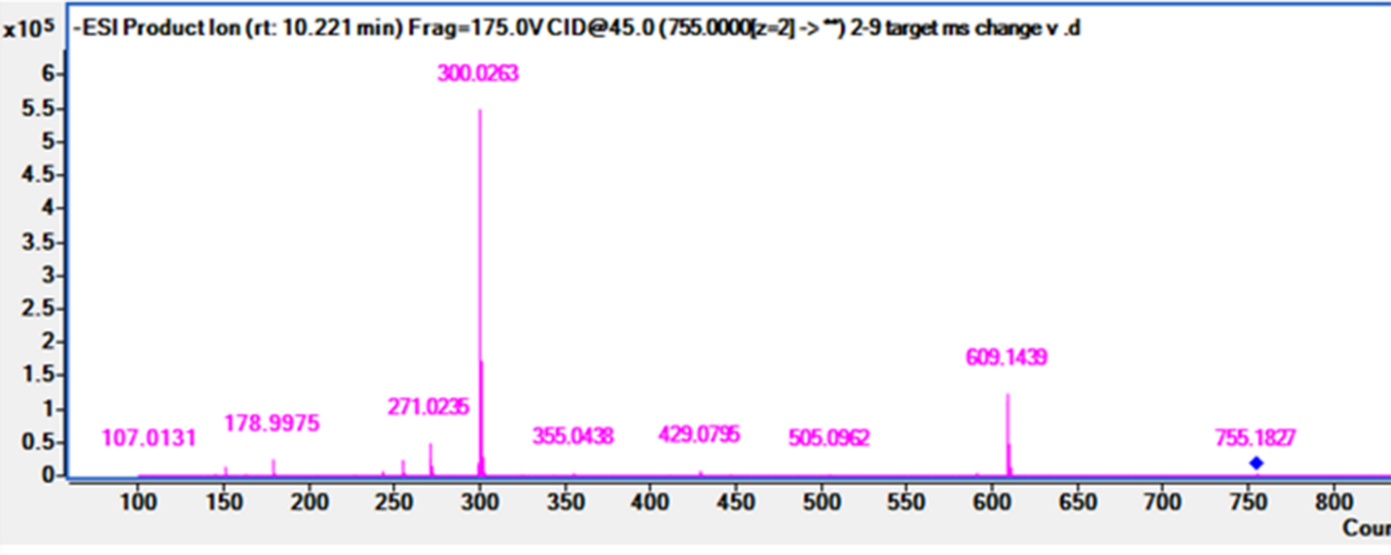
**

Figure S11: The mass spectrogram of Compound 11

**
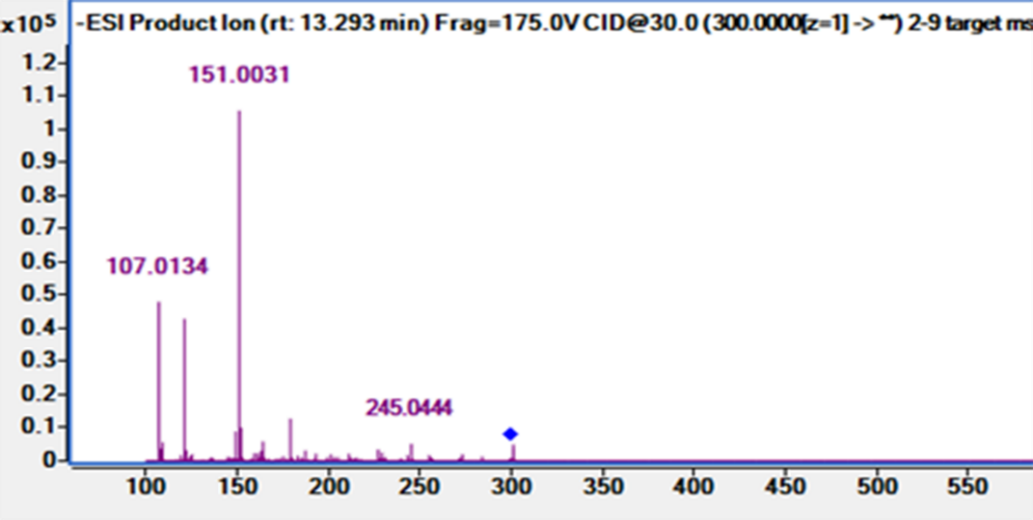
**

Figure S12: The mass spectrogram of Compound 12

**
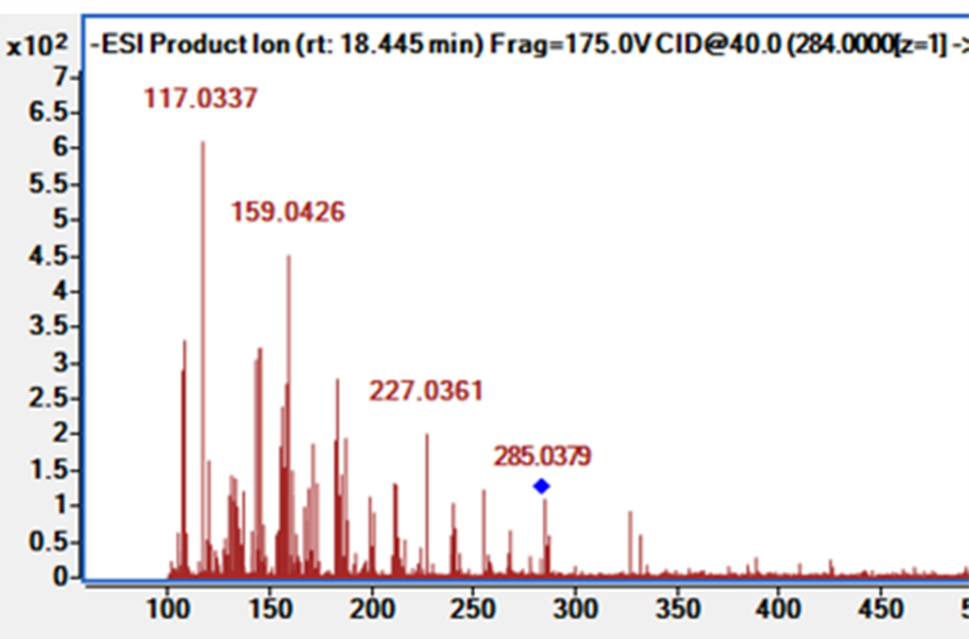
**

Figure S13: The mass spectrogram of Compound 13

**
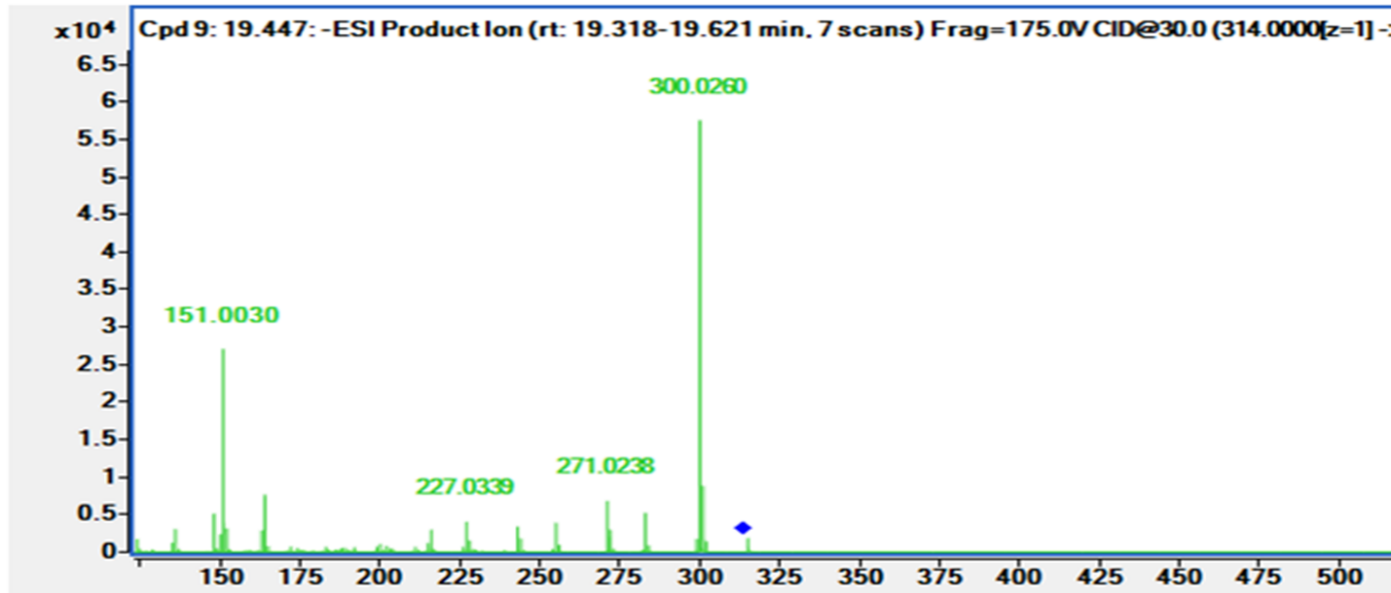
**

Figure S14: The mass spectrogram of Compound 14
